# Making the Invisible Companion of People with Dementia Visible in Economic Studies: What Can We Learn from Social Science?

**DOI:** 10.3390/healthcare9010044

**Published:** 2021-01-05

**Authors:** Kim-Huong Nguyen, Tracy Comans

**Affiliations:** Centre for Health Services Research, The University of Queensland, Herston, QLD 4006, Australia

**Keywords:** economic evaluation, dementia, dyadic perspective, interaction, correlation, regression, path analysis, health outcomes, quality of life, scoping review

## Abstract

The dyadic perspective is important to understand the mutual influence and interdependence of both the person living with dementia and their care partner. This perspective is routinely adopted in social research programs for dementia and many dyadic interventions have been developed. However, economic evaluation and modelling to date has often failed to incorporate caregivers’ perspectives, and their respective costs and outcomes while giving care for the person with dementia. On the occasions that this has been done, caregivers were represented as “informal costs” associated with dementia. This limited perspective cannot incorporate two-way interactions of the dyad in economic evaluations of dementia programs. This paper provides an overview of the possible interactions between people living with dementia and care partners as discovered in social science literature in the past 20 years. We demonstrate the strength of the relationships and discuss strategies for incorporating the dyadic perspective in economic evaluations of dementia programs in the future.

## 1. Introduction

People with dementia who live in the community often rely on assistance from caregivers to perform daily functions. Caregivers are family and/or friends (informal), or paid care workers (formal). Assistance and care activities range from safety and well-being support to personal and instrumental activities of daily living [1]. It was estimated that, on average, caregivers spent close to 4 hours per day providing assistance and care for the person with dementia [2]. A global cost of dementia study estimated that close to a half of the economic cost was in some form of informal care. That was US$402 billion, out of US$948 billion of the total economic cost of dementia, in 2016, globally [3]. It would require considerably more economic resources if all informal care was replaced by skilled and unskilled paid care services. For instance, Chari et al. [4] estimated that the paid services for elder-care in the US (all types of conditions) would have amounted to 1.6 times the opportunity cost of informal care (i.e., $863 billion versus $522 billion informal elder-care).

Research on the dyadic relationship of people with dementia and their carers has been conducted in different disciplines, from psychology to nursing and social work. This consists of both trials and prospective cohort studies, and literature reviews. Research themes ranged from the impacts of dyadic interventions on health and quality of life outcomes, to the burden of caregiving and its impact on mental and physical health, and quality of life of the caregivers, to the dyad relationship, and coping strategies [5,6,7,8,9]. Caregiving is often found to have negative health impacts on the informal caregivers (including poor cardiovascular health and depression symptoms), both in the short and long term [10,11]. This has implications from both the individual’s (health outcomes, quality of life, medical expenditure and financial security) and society’s perspective (lower productivity of the workforce, and future morbidity burden) [12,13,14,15].

In the economic evaluation literature, the relationship between people with dementia and their caregivers was summarised as “informal care service” provided by the caregivers. Often presented as a cost item, it was quantified as the values of the time that the caregivers spent on looking after the person with dementia (i.e., opportunity cost) [16]. No economic studies quantified the gain or loss of health outcomes (of the person with dementia) induced by the caregivers’ availability and competency. Neither did any study quantify the value of health loss by the caregivers despite a sizable literature confirming the negative impact of caring on long-term health outcomes.

The absence of these economic outcomes and costs in an economic evaluation has two implications. First, the full economic cost would not be quantified. For instance, a new intervention that relieves caregivers of 10 hours of care duties per week so they can return to work part-time, or look after their own mental and physical health requires additional resources (professional care-workers, and vehicles to travel to the clients). The caregivers’ gain can lead to improved care quality, leading to better quality of life for the person with dementia, and caregivers’ ability to care for a longer period of time (i.e., delayed entry to nursing home). If the economic study assumed no change in the health outcomes of the people with dementia who received the service, and only accounted for the opportunity cost of the caregivers, then some of the values gained from “reduced informal care” would not be factored in the calculation. For a given outcome (of the person with dementia), the new intervention would look relatively more expensive than the status quo (without the intervention) due to the increased resources required. This might lead to an erroneous conclusion that the new intervention is not cost-effective.

Second, the trade-offs of outcomes (and costs) between caregivers and people with dementia would be masked. Take the example above, if the person with dementia pays for the services, the transfer of economic benefits (or financial losses) from the person with dementia to the caregivers would be much larger when all economic costs and outcomes are taken into account. If there is a centralised payer for care services (instead of individual patients paying out of pocket), knowledge about such information would highlight the cost-shifting between public and private funding, and between community care and nursing homes. This might lead to a more efficient and equitable cost-sharing mechanism between the public budget, person with dementia, and their caregivers. In an economic evaluation, it is as important to know about the distribution of outcomes and costs as it is to know about whether or not an intervention leads to net gain overall.

This scoping study is an attempt to bring the rich knowledge discovered within the dementia dyad relationship literature to economic evaluation studies. It aims to explore how the impacts of co-dependency and reciprocal effects of dementia dyads have been measured and quantified, and suggests how this knowledge can be incorporated for informing the economic evaluation of dementia interventions.

## 2. Materials and Methods

We undertook a scoping review using the methodology outlined by Aksey and O’Malley [17] and Levac et al. [18]. A systematic literature review, and meta-analysis, were not considered in this study. We wanted to explore the themes that naturally emerged from this literature, rather than focusing on critically appraising and synthesising evidence (as in a systematic review) [19]. This will serve as the precursor for future systematic reviews that focus on the conceptual relationship between specific outcomes of people with dementia and their caregivers, or investigating conflicting results in the literature.

This scoping review consists of five stages:

- Stage 1—Identify the research questions for scoping study:

We want to know what the literature found about the two-way correlations between disease progression in people with dementia and their (informal) caregivers’ health and well-being. More specifically, (i) what are the impacts? (ii) how were they measured? (iii) what was the impact size? and (iv) how do we reflect these into an economic evaluation study?

Since the review focuses on “impact measures”, we only included quantitative studies, i.e., those containing statistical analyses such as regression and/or correlation. Additionally, we were only interested in studies that investigated two-way correlations rather than one-way (i.e., those studying only impacts of dementia progression on the caregivers’ outcomes or impacts of caregivers’ capacity on dementia progression and other outcomes of people with dementia).

- Stage 2—Identify relevant studies:

The literature search was conducted in line with the Preferred Reporting Items for Systematic Review and Meta-Analyses (PRISMA) guidelines [20]. The search was applied to literature available up to October 2020, in the electronic databases of MEDLINE, Embase, and PubMed. We also checked reference lists of identified publications and hand-searched key journals and relevant websites (organisations, conferences, networks) for additional articles meeting the inclusion criteria.

- Stage 3—Study selection using pre-determined inclusion criteria:

Inclusion criteria were studies that (i) included outcomes of both people with dementia and of caregivers, (ii) analysed the outcomes by both members of the dyads using statistical methods, and (iii) reported quantitative results of the analyses, from which correlation and/or regression coefficients could be extracted.

We excluded studies that (i) investigated the impacts of interventions on people with dementia and their caregivers, or (ii) established the statistical relation amongst health outcomes or wellbeing of each member of the dyad (only caregivers, or only people with dementia), or (iii) had sample size smaller than 50 dyads, or (iv) investigated family caregivers in the long-term care context (i.e., nursing homes or residential aged care facilities). We did not define any exclusion criteria on the type of outcome measures to allow a natural emergence of themes in Stage 5.

The identification of included studies was undertaken by two independent reviewers, who initially screened the titles and abstracts for eligibility using CoEvidence (https://www.covidence.org/). A study was included if both reviewers agreed that it met all the inclusion criteria. Dissimilarities in reviewers’ conclusion were resolved by discussion and/or examining the full article. Full texts of all included studies were reviewed independently by both reviewers. Resulting citations and reference lists in these reviewed studies, studies that published systematic reviews, and meta-analyses were examined to ensure that no eligible studies were missed. Both reviewers systematically and independently documented the properties of the included studies.

- Stage 4—Charting the data:

We synthesised and interpreted the data by charting and sorting quantitative information according to key analytical features and themes. The data were entered onto a “data charting form”, which is a mixture of general information about the study (author, year, country) and specific information related to the research question, outcome measurements for the caregivers and people with dementia, data type, sample size, statistical methods, and main findings.

- Stage 5—Collating, summarising, and reporting results:

From the charted data, we grouped the similar findings and extracted the common themes in the literature to answer the four research questions formulated in stage 1. During this process, we attempted to map all the bilateral impacts discussed in this literature using a pathway map.

First, all the outcomes documented in this literature were grouped into categories. We did not limit the number of categories or the number of measures to be included in each category. We reported precisely and truthfully what was used in this literature, to understand the diversity in outcomes and measurements, and let the themes emerge naturally.

Second, connections (paths) were drawn between each pair of outcomes if their associations or correlations were reported to be statistically significant. We expected many pairs to have repeated connections while others might only have one or two. If the correlation direction between two outcomes were consistent across studies, either positive or negative, the connection was given that specific direction. If they were found to be opposite by different studies, but statistically significant in each of those studies, we did not assign a direction.

While interested in impact sizes and directions, we made no attempt to conduct meta-regression analysis or assign “weight” to the evidence in this literature. This is consistent with the general approach of a scoping study in which quality assessment was not the main objective. However, we provided some judgement on whether this literature provided robust or generalisable findings that can be used in economic evaluation studies.

## 3. Results

**Figure 1:**
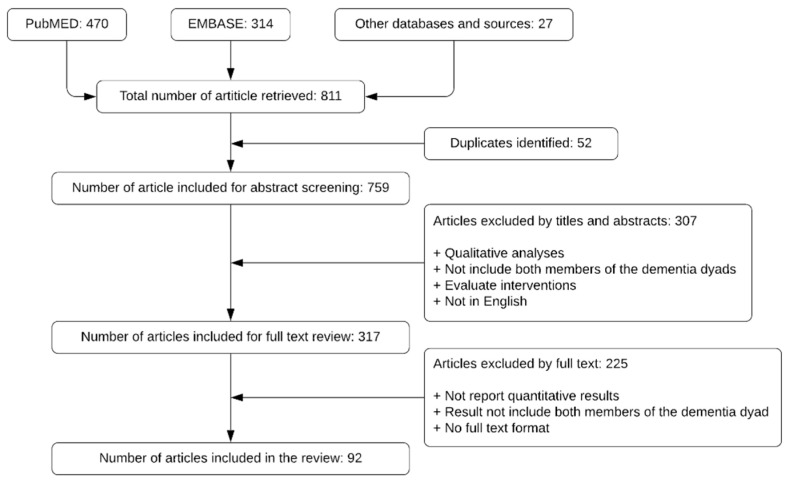
Literature screening and selection, following the Preferred Reporting Items for Systematic Review and Meta-Analyses (PRISMA) guideline.

The systematic literature search resulted in more than 600 entries. From stages 1 to 3, we arrived at a final list of 92 studies for stage 4. The majority of studies excluded were either qualitative research or evaluating interventions for people with dementia or their caregivers, or focusing on one member of the dementia dyad only (either the person with dementia, or their caregivers, but not both, at the same time). The search result is presented in Figure 1, and the full list of studies included in the review can be found in the Supplementary Materials.

On average, there were about six studies published per year between 2005 and 2020. There was a strong trend of increased number of studies in more recent years. The majority of studies were undertaken in European countries, some of which are multi-country studies [21,22,23,24], followed by the Asia-Pacific and the US. About 80 percent of the studies used cross-sectional data (one time point). The majority of the longitudinal studies had between two and four time points. The target population was mixed, covering people with mild cognitive decline and those having any form of dementia (70 percent of studies) or those with a diagnosis of Alzheimer’s disease (about 30 percent of studies).

Research questions of this literature can be loosely grouped into three categories. First, investigating the correlations between health outcomes and/or quality of life of people with dementia and their caregivers. Second, understanding the determinants of outcomes of the person with dementia. Third, searching for determinants of outcomes of caregivers. More than a half of studies focused on outcomes of caregivers, and the remainders split roughly equally to the first and second categories. The determinants often included characteristics of both members of the dyads, measures of health outcomes, and/or “mediators” of those outcomes. For the caregivers, the outcomes of interest were care burden, quality of life, mental and physical health; the mediators often included relationships with the person with dementia, coping style, sleep, and care duration. For the person with dementia, standard measures were cognitive capacity, behaviour and psychological health, activities of daily living, and quality of life; individual characteristics included diagnosis type, time since diagnosis, and comorbidity.

For the person with dementia, all the outcome measures were sorted into six groups (domains): three clinical outcome domains, quality of life, health-related outcomes and individual characteristics. There were multiple instruments used for each domain, especially the clinical outcomes. For example, “cognitive capacity” could be measured by the MMSE (Mini-Mental State Examination), CDR (Clinical Dementia Rating), MoCA (Montreal Cognitive Assessment), CAMCog (Cambridge Examination for Mental Disorders—cognitive part); “behaviour and psychological health” could be measured by the Neuropsychiatric Inventory (NPI), BEHAVE-AD (Behavioural Pathology in Alzheimer’s Disease), BDSD (Behaviour Disturbance Scale in Dementia); “activities of daily living” could be captured by ADL (activities of daily living) and iADL (instrumental activities of daily living), or by the FAQ (Pfeffer Functional Activity).

For the caregivers, we grouped all the outcome measures into six groups, of which two capture mental and physical health. Burden, being a top researched topic in this literature, was given its own domain. Similar to the outcome measures for people with dementia, there were multiple instruments used for each outcome domain of the caregivers. For instance, burden was measured by the CBI (Caregiver Burden Inventory) or ZBI (Zarit Burden Index); their depression and mental health was measured by the BAI (Beck Anxiety Inventory), GADS (Goldberg Anxiety and Depression Scale), CES-D (Center for Epidemiologic Studies Depression Scale) or NPI-Distress; quality of life was measured by WHO-QOL (World Health Organisation quality of life instrument), EQ5D, or SF-12 (Short-Form 12 items). The full list of outcome measures used are summarised in Table 1.

Most studies applied standard statistical techniques for social science research. These ranged from statistical tests (analysis of variance—ANOVA, and two-sample mean tests, with and without adjustment for additional covariates) to correlation analyses (from univariate to multivariate). A small number of studies used sophisticated regression models such as growth mixture model, generalised linear model and structured equation system [25,26,27,28] (these studies are discussed in more detail below). About 40 percent of the studies presented detailed analyses: starting from data exploration and statistical tests of individual variables, to regression analyses that aimed at establishing correlations or causation between interested outcomes, adjusting for participant characteristics and relevant confounders. All studies considered age and gender in their analyses, and a small number of studies extended these to living arrangement, financial situation, and years into the disease (time from diagnosis or symptom).

**Figure 2:**
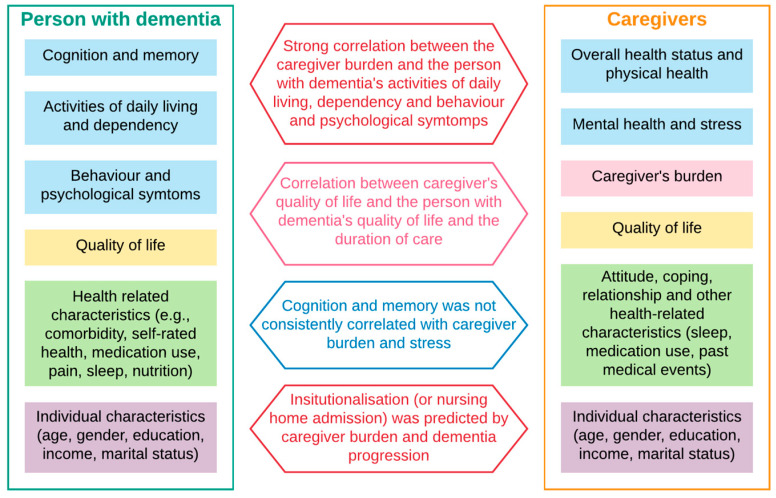
Summary of correlations between outcomes of caregiver and person with dementia.

Figure 2 summarise the main findings in the literature. First, high dependency, low activities of daily living, severe behaviour and psychological symptoms were strongly associated with higher caregiver burden, which in turn determined the higher likelihood of nursing home admission (requiring full-time care or institutionalisation). Across studies that used different techniques, either correlation or regression analyses, and different instruments to capture those domains (activities of daily living and dependency, behaviour and psychological symptoms and caregiver burden), statistically significant evidence was found to support this. We captured the direction of impact and ignored the effect sizes because of the high heterogeneity of instruments used within each domain.

Second, caregiver quality of life correlated with the care duration, the person with dementia’s behaviour and psychological symptoms and quality of life. Significant moderating factors included caregiver coping style, personality, living environment, and quality of the dyad relationship. The findings were evident across studies, despite a wide variety of instruments used to measure quality of life (of the caregiver) and behaviour and psychological symptoms of the person with dementia. Similar to the previous findings, we noted the direction, but not the magnitude, of impacts.

Third, while cognitive impairment is the signature symptom of dementia, its severity was not consistently correlated with caregiver burden and poor psychological health (stress, anxiety, depression). However, it was a strong predictor of time to nursing home admission (requiring full-time care/institutionalisation).

**Figure 3:**
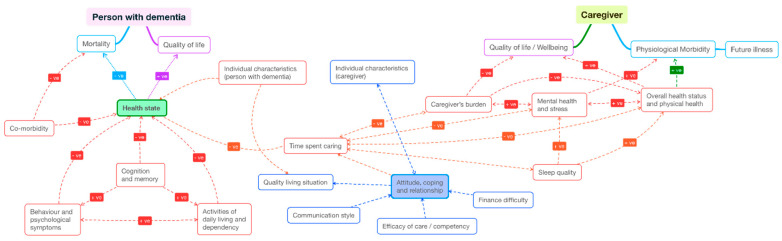
Mapping of interactions between outcomes of caregiver and person with dementia.

At the end of step 5, we mapped the quantitative findings of this literature in a pathway map (Figure 3). All factors and outcomes analysed in the literature were noted and sorted loosely into clinical symptoms, demographic characteristics, mediating factors (caregiver’s competency, coping strategies, care duration and intensity, sleep quality) and “ultimate outcomes” (quality of life, wellbeing, morbidity, and mortality). The clinical symptoms of people with dementia consist of three domains specified in Table 1 and Figure 2 (“cognition and memory”, “behaviour and psychological symptoms”, and “activities of daily living and dependency”). The clinical symptoms of caregivers include the two domains of “overall health status and physical health”, and “mental health and stress”. The direction of impact or causality reflected hypotheses posed in the literature, as partly summarised in Figure 2. Each correlation that was found to be statistically significant (between each pair of variables) was given a path, being positive (+ve) or negative (−ve) or inconclusive (neither +ve or −ve). A path was defined as “inconclusive” when there was mixed evidence (yet statistical significance) by different studies.

While most studies emphasised “association” and “relationship” between outcomes and characteristics of dementia dyads, a small number of studies attempted to establish the causality pathways, where certain behaviours or intermediate outcomes influence the primary outcome of interest. These studies visually mapped the pathways and estimated the correlation coefficients for each path. We discuss these findings in more detail below.

Cooper et al., (2008) applied structural equation modelling on the LASER-AD dataset (a longitudinal study of people with Alzheimer’s disease and their caregivers from metropolitans and semi-rural areas in London and South-East Region of England) [25]. The authors aimed to establish the causal relationship between anxiety and coping strategies of the caregivers. The researchers interviewed 129 caregivers of people with dementia at baseline, of whom 93 were re-interviewed one year later. Health outcomes of the people with dementia whose caregivers were interviewed were extracted from the LASER-AD study. The study started with data exploration and correlation analyses of key outcome variables of the two time periods, (including caregiver burden, anxiety, coping strategies) followed by the structural equation modelling where variables with reasonable correlation coefficients entered the estimated equations. The hypothesis that coping mediated the relationship between burden and anxiety, the authored sequentially added and removed coping, burden and anxiety of both time periods, to test the direction of causality. They concluded that effective coping strategies led to lower burden and anxiety (i.e., not because of lower anxiety, the caregiver found more effective coping strategies).

Morgan et al., (2013) also used a path model to establish the cause of aggressive behaviours in people with dementia [27]. The authors developed a survival model that included a large number of key outcomes of the dementia dyads (non-aggressive physical agitation, dementia severity, depression, pain, caregiver burden and mutuality) using a longitudinal dataset of 171 dyads (with 7 time points). Their model simultaneously estimated the regression and survival components, enabling the calculation of both indirect and direct relationships between interested outcomes. The study established the direct causation between elevated risk of agitation (by the person with dementia) and high level of burden at baseline, deteriorated dyad relationship, increased non-aggressive physical agitation and pain by the person with dementia. Meanwhile, baseline dementia severity and depression were only the indirect cause of agitation.

Garre-Olmo et al., (2016) was another study that attempted to establish causal pathways, using a system of equation approach [26]. They developed a path model to explain caregiver burden using contextual factors (including caregivers age and gender, residential/living situation and number of caregivers involved)*,* primary stressors (person with dementia disease severity, neuropsychiatric symptoms and dependency), and secondary stressors (employment status of the caregiver). Similar to Cooper et al. [25], they started with a series of bivariate analyses of the demographic, clinical characteristics and outcomes of the dementia dyads, from which the path model was developed to reflect the statistically significant correlations. The authors found that dependency (of the person with dementia), sociodemographic characteristics of the caregivers and their distress level were the main direct predictors of the caregiver burden. Dependency was influenced by the severity of neuropsychiatric symptoms and disability caused by dementia, and neuropsychiatric symptoms had an important impact on caregiver distress.

Lastly, Zahir et al., (2020) explored the mediating effect of caregiver “objective attitude” on their strain and relationship with the person with dementia using a structural equation model [28]. Similar to Garre-Olmo et al. [26], they used cross sectional data of 215 dementia dyads. Objective attitude was measured by an 18-item instrument developed by the authors, while other outcomes were assessed using standard instruments (Caregiver Strain Index, Relationship Closeness Scale, Clinical Dementia Rating). The study found that the “objective attitude” did not protect caregivers against strain and was associated with decreased relationship closeness between them and the person with dementia. In other words, adopting the “objective attitude” might not be an effective response strategy to improve health and relationship outcomes.

## 4. Discussion

In this paper, we reported findings from a comprehensive scoping review of the quantitative literature that examine the quantifiable outcomes of the co-dependency relationship by dementia dyads. The aim is to use this body of knowledge to inform future economic evaluations of dementia interventions. Following the standard approach of a scoping review, we presented an overview of all materials and summarised the main themes and findings of the literature.

As expected, this is a diverse and rich literature. The studies were conducted under different disciplines: clinical, neuro-psychiatrics, dementia-specific, nursing, social work, gerontology and psychogeriatrics, and multidisciplinary. While the diversity of theoretical approaches adds to the strength of conclusions, this is tempered by the complex subject matter. There are many potential co-founders outside the control of the researchers/experimenters and different measures to capture the same outcomes. For instance, we found that there were 15 instruments used to measure the severity of cognitive impairment, of which MMSE was the most popular (used in 80% of studies) followed by CDR (used in 30% of studies). Similar observations were made for behaviour symptoms (9 instruments) and physical health and dependency (15 instruments). The majority of studies used more than one instrument to measure each of those domains. While this practice strengthened the validity of findings between clinical outcomes of people with dementia and other outcomes of interest (of both caregivers and people with dementia), heterogeneity in outcome measures makes synthesising the quantitative evidence (effect size) challenging. The effect size (and its direction) is crucial in modelling the interaction between the people with dementia and their caregivers if the objective is to quantify the economic impacts of interventions for the dementia dyads.

In all studies, authors acknowledged data limitations and its impact on the choice of quantitative method, and subsequently the validity and generalisability of their findings. Data limitations included (i) incomplete set of relevant outcomes and factors that allowed them to test for different mediators and causal pathways, (ii) proxy completion, that is caregivers completing outcome surveys on behalf of the people with dementia, and (iii) the lack of longitudinal data with decent sample size that allows for the incorporation of non-observable or non-measurable factors in the dyad relationship. Of the 11 studies using longitudinal data, only three had a sample size larger than 500 pairs of dementia dyad [29,30,31]. Both studies that attempted to establish causations using longitudinal data [25,27] had a sample size smaller than 200, with limited number of outcomes and dyad characteristics. Seven out of nine studies with a large sample size (>1000) were cross-sectional. It was inevitable that correlations, not causation, between key outcomes, were established. Additionally, the robustness of estimated effect size and direction from a small cross-sectional sample was often unwarranted. If findings in this literature are to be used as evidence to inform the development of policies and interventions targeting dementia dyads, larger longitudinal data with comprehensive and standardised outcome measures is required.

Despite its richness in themes and insights about a complex relationship of the resource-intensive disease, we found that the quantitative findings in their current form were not readily usable for economic evaluation studies. We were able to extract the main themes and developed a pathway map that presented all the correlations found in the literature. This map is useful to guide economists when selecting relevant outcomes to formulate their evaluation, and how to present the interactions of the dyads. However, it was not possible to assign a coefficient to each path, due to the wide variation of estimated results and the lack of robust causality. Economic evaluations using Markov models or hybrid simulation approaches rely on not only the direction but also the strength of different pathways.

With the global aging population, and dementia being a leading cause of death in people aged 65+, undoubtedly the body of literature investigating the bi-directional impacts between people with dementia and their family/friend caregivers will continue to grow. Future studies can build upon the limitations of past studies. We recommend the following approaches.

First, a standardised approach across study instruments used to measure the same outcome. As noted above, the heterogeneity of instruments used in this literature leads to difficulty in comparison and synthesising quantitative evidence. Nonetheless, the persistent evidence of strong correlation between certain outcome domains (such as people with dementia’s behaviour and psychological symptoms and caregiver burden) indicated that different instruments were successfully capturing the clinical fundamentals of the health condition and their underlying relationships. A clinical diagnosis of dementia is often complex, and researchers sometimes do not gain access to clinical measures of dementia such as biomarkers and PET scans. The use of symptom-based instruments can be the cost-effective second best. Currently, many outcome instruments are well validated and used widely in both clinical and social studies of dementia. They should form a core set of outcome measures for dementia and their caregivers, complemented by additional instruments that are deemed necessary. It is encouraging to see the core outcome movement taking place across many disease domains, including dementia (more information can be found at https://www.comet-initiative.org/studies/details/677 and https://www.ichom.org/portfolio/dementia/). These will increase the possibility of evidence synthesis and improve the validity of findings across studies.

Second, applications of advanced statistical methods to establish causation between outcomes and mediators. The lack of large longitudinal data and applied statistical methods that could establish causality leads to weak evidence for intervention and policy formulation. In the literature review supporting their study on health effects of caregiving, Coe et al., (2009) also found that the majority of studies used cross-sectional data and/or failed to account for endogeneity between health and informal care, especially in the long term. [32] While resource intensive, registries and large cohort studies for major diseases have becoming more feasible in the last 20 years, thanks to advancement in information technologies, improved storage capacity and data collection methods. The new era of big data means there are innovations in this space that can be brought into the collection of data from people with dementia and their caregivers.

Lastly, using advanced modelling approaches to test for potential causal pathways. While the causality between certain outcomes and mediators have not yet been confirmed by the literature, it is possible to use hybrid simulation models to form and test those hypotheses. Hybrid simulation models have become more accepted in health care operation and implementation research. Their current applications concentrate around resource allocation studies (e.g., queuing models using discrete event simulation) and transmittable diseases (e.g., pandemic and outbreaks of communicable diseases). The development of those models in chronic disease to understand the disease burden, health services required, and its economic impacts will enrich the literature and potentially revolutionise the way health services and polices are designed and implemented.

## 5. Conclusions

Family caregiving is a dynamic process, and both care recipient and caregiver constantly adjust to each other’s changes, which impacts on dementia progression, health outcomes and quality of life of both. When this process can be better understood and embedded in future economic studies, interventions and care practices that strike the efficient and equitable trade-off balance will be identified and rapidly translated into practice to improve health outcomes and quality of life for the dementia dyads.

## Figures and Tables

**Table 1:** Outcome measures and instruments used in the literature.

Person with Dementia	
**Cognition and memory**	Mini Mental State Examination (MMSE), Cambridge Cognition Examination (CAM-Cog), Clinical Dementia Rating (CDR), Montreal Cognitive Assessment (MoCA), Dementia Rating Scale (DRS), Global Deterioration Scale (GDS), Alzheimer’s Disease Assessment Scale–Cognitive Subscale (ADAS-Cog), Clock Drawing Test (CDT), Cognitive Abilities Screening Instrument (CASI), Screening instrument for dementia consisting of 5 subtests (DemTect)
**Activities of daily living and dependency**	Barthel Index of Activities of Daily Living, Physical Self-maintenance Scale (PSMS), Instrumental Activities of Daily Living (IADLS), Nurses’ Observation Scale for Geriatric Patients (NOSGER), Alzheimer Disease Cooperative Study Activities of Daily Living Inventory (ADCS-ADL), Allen battery, Basic Activities of Daily Living (BALDs), Blessed Functional Activity Scale score (Blessed FAS), Dependency scale, Caregiver Assessment of Function and Upset (CAFU), Resources for Enhancing Alzheimer’s Caregiver Health (REACH), Disability Assessment for Dementia (DAD), Pfeffer Functional Activity (FAQ), Katz Activities Scale (KAS), Functional Autonomy Measurement System (SMAF), Dependence Scale (DS)
**Behaviour and psychological symptoms**	Agitation, Behavioral Pathology in Alzheimer’s Disease (BEHAVE-AD), Dementia Behaviour Disturbance Scale (DBDS), Behaviour Frequency, Cornell Scale for Depression in Dementia (CSDD), Cohen-Mansfield Agitation Inventory (CMAI), Neuropsychiatric Inventory (NPI), Brief Psychiatric Rating Scale (BPRS), NeuroPsychiatric Symptoms of dementia (NPS), Hamilton Rating Scale for Depression (HRSD), Geriatric Depression Screening (GDS)
**Quality of life**	European Quality of Life 5-Dimension scale (EQ5D), Quality of Life in Alzheimer’s Disease (QoL-AD), Quality of Life (QoL) one question
**Health-related characteristics**	Cumulative Illness Rating Scale (CIRS), Charlson Comorbidity Index (CCI), Dyadic Coping Inventory (DCI), Clinical Global Impression (CGI), Incalzi Comorbidity Index, Number of medical conditions, Physical comorbidity, Self-rated health, Time since onset of dementia/years of symptoms, Dementia diagnosis, Medication use, Use of psychotropic medicine, Sleep quality, Nutrition level, Philadelphia Pain Intensity Scale (PIS), Level of care required
**Individual characteristics**	Age, Gender, Education level, Race, Marital status, Number of children, Living arrangement, Financial situation, Income, Insurance, Quality of Marriage Index (QMI)
**Caregiver**	
**Overall health status and physical health**	Self-reported clinical problems, Physical health, Health status, Self-reported health, Medical conditions
**Mental health and stress**	Beck Depression Inventory (BDI), Beck Anxiety Inventory (BAI), Center for Epidemiologic Studies-Depression (CED-D), Frustration of caregiving subscale from Resources for Enhancing Alzheimer’s Caregiver Health II trial (Frustration REACH II), Generalized Anxiety Disorder 7-item scale (GAD-7), Patient Health Questionnaire (PHQ-9), Geriatric Depression Screening (GDS), Felt Expressed Emotion Rating Scale (FEERS), Expression of Interest (EOI), Perceived Stress Scale 14-item scale (PSS-14), 12-item General Health Questionnaire (GHQ), Goldberg Anxiety and Depression Scale (GADS), Hamilton Depression Rating Scale (HDRS), State-Trait Anxiety Inventory (STAI), Neuropsychiatric Inventory Distress Scale (NPI-D), Beck Hopelessness Scale (BHS)
**Caregiver’s burden**	Lawton Caregiving Burden Scale, Caregiver Burden Inventory (CBI), Burden Scale for Family Caregivers (BSFC), Burden interview, Zarit Burden Interview (ZBI), Self-Rated Burden scale (SRB), Self-Perceived burden from Informal Care (EDIZ: ‘Ervaren Druk door Informele Zorg’)
**Quality of life**	The 12-Item Short Form Health Survey (SF-12), 36-Item Short Form Health Survey (SF-36), 15D health-related quality of life instrument (15D), Visual analogue scales (VAS), Lawton caregiving satisfaction scale, European Quality of Life 5-Dimension scale (EQ5D), Health Status Questionnaire-12 (HSQ-12), Life satisfaction, Quality of Life (QoL) one question, Quality of Life in Alzheimer’s Disease (QoL-AD), Schedule for the Evaluation of Individual Quality of Life (SEIQoL), World Health Organization Quality of Life (WHOQOL)
**Attitude, coping, relationship and other health-related characteristics**	Sleep interruption, Pittsburgh Sleep Quality Index (PSQI), Positive and Negative Affect Schedule (PANAS), Antidepressant medication use, Battery of Generalized Expectancies of Control Scales (BEEGC-20), Caregiver Reaction Assessment (CRA), Dyadic Coping Inventory (DCI), Quality of Marriage Index (QMI), Neuroticism sub-scale of the Eysenck Personality Questionnaire (EPQN scale), Couple’s Respect Questionnaire (CRQ), Relationship quality (with person with dementia), Past life events, History of emotional problems, Mutuality scale (relationship), Positive Aspect of Care (PAC), Adaptation—Partnership—Growth—Affection—Resolve (APGAR index), Social Support Rating Scale (SSRS), Negative communication style, Sexual Experience and Satisfaction (QSES), Relationship closeness scale, Resilience scale
**Individual characteristics**	Age, Gender, Race, Education level, marital status, Kinship (with person with dementia), Employment, Income, Living arrangement, Care duration, Shared care, Financial situation, Number of children

## Data Availability

Data charted from the literature can be provided upon requests. The full list of studies reviewed can be found in the Web Appendix accompanying this manuscript.

## Supplementary Materials

The following are available online at https://www.mdpi.com/2227-9032/9/1/44/s1, Web Appendix.

## References

[1] Laver K., Milte R., Dyer S., Crotty M. (2017). A Systematic Review and Meta-Analysis Comparing Carer Focused and Dyadic Multicomponent Interventions for Carers of People with Dementia. J. Aging Health.

[2] Wimo A., Winblad B., Jönsson L. (2007). An estimate of the total worldwide societal costs of dementia in 2005. Alzheimers Dement..

[3] Xu J., Zhang Y., Qiu C., Cheng F. (2017). Global and regional economic costs of dementia: A systematic review. Lancet.

[4] Chari A.V., Engberg J., Ray K.N., Mehrotra A. (2015). The Opportunity Costs of Informal Elder-Care in the United States: New Estimates from the American Time Use Survey. Health Serv. Res..

[5] Black W., Almeida O.P. (2004). A systematic review of the association between the Behavioral and Psychological Symptoms of Dementia and burden of care. Int. Psychogeriatr..

[6] Del-Pino-Casado R., Rodríguez Cardosa M., López-Martínez C., Orgeta V. (2019). The association between subjective caregiver burden and depressive symptoms in carers of older relatives: A systematic review and meta-analysis. PLoS ONE.

[7] Monteiro A.M.F., Santos R.L., Kimura N., Baptista M.A.T., Dourado M.C.N. (2018). Coping strategies among caregivers of people with Alzheimer disease: A systematic review. Trends Psychiatry Psychother..

[8] Quinn C., Toms G. (2019). Influence of Positive Aspects of Dementia Caregiving on Caregivers’ Well-Being: A Systematic Review. Gerontologist.

[9] Stall N.M., Kim S.J., Hardacre K.A., Shah P.S., Straus S.E., Bronskill S.E., Lix L.M., Bell C.M., Rochon P.A. (2019). Association of Informal Caregiver Distress with Health Outcomes of Community-Dwelling Dementia Care Recipients: A Systematic Review. J. Am. Geriatr. Soc..

[10] Watson B., Tatangelo G., McCabe M. (2019). Depression and Anxiety among Partner and Offspring Carers of People with Dementia: A Systematic Review. Gerontologist.

[11] Xu X.Y., Kwan R.Y.C., Leung A.Y.M. (2020). Factors associated with the risk of cardiovascular disease in family caregivers of people with dementia: A systematic review. J. Int. Med. Res..

[12] McGarry K.M. (2006). Does Caregiving Affect Work? Evidence Based on Prior Labor Force Experience. Health Care Issues in the United States and Japan.

[13] Heitmueller A. (2007). The chicken or the egg? Endogeneity in labour market participation of informal carers in England. J. Health Econ..

[14] Heitmueller A., Inglis K. (2007). The earnings of informal carers: Wage differentials and opportunity costs. J. Health Econ..

[15] Fujihara S., Inoue A., Kubota K., Yong K.F.R., Kondo K. (2019). Caregiver Burden and Work Productivity among Japanese Working Family Caregivers of People with Dementia. Int. J. Behav. Med..

[16] Nguyen K.-H., Comans T.A., Green C. (2018). Where are we at with model-based economic evaluations of interventions for dementia? A systematic review and quality assessment. Int. Psychogeriatr..

[17] Arksey H., O’Malley L. (2005). Scoping studies: Towards a methodological framework. Int. J. Soc. Res. Methodol..

[18] Levac D., Colquhoun H., O’Brien K.K. (2010). Scoping studies: Advancing the methodology. Implement. Sci..

[19] Munn Z., Peters M.D.J., Stern C., Tufanaru C., McArthur A., Aromataris E. (2018). Systematic review or scoping review? Guidance for authors when choosing between a systematic or scoping review approach. BMC Med. Res. Methodol..

[20] Moher D., Liberati A., Tetzlaff J., Altman D.G., PRISMA Group (2009). Preferred reporting items for systematic reviews and meta-analyses: The PRISMA statement. BMJ.

[21] Alvira M.C., Risco E., Cabrera E., Farré M., Hallberg I.R., Bleijlevens M.H., Meyer G., Koskenniemi J., Soto M.E., Zabalegui A. (2015). The association between positive-negative reactions of informal caregivers of people with dementia and health outcomes in eight European countries: A cross-sectional study. J. Adv. Nurs..

[22] Haro J.M., Kahle-Wrobleski K., Bruno G., Belger M., Dell’Agnello G., Dodel R., Jones R.W., Reed C.C., Vellas B., Wimo A. (2014). Analysis of burden in caregivers of people with Alzheimer’s disease using self-report and supervision hours. J. Nutr. Health Aging.

[23] Reed C., Belger M., Dell’Agnello G., Wimo A., Argimon J.M., Bruno G., Dodel R., Haro J.M., Jones R.W., Vellas B. (2014). Caregiver Burden in Alzheimer’s Disease: Differential Associations in Adult-Child and Spousal Caregivers in the GERAS Observational Study. Dement. Geriatr. Cogn. Disord. Extra.

[24] Sousa M.F.B., Santos R.L., Turró-Garriga O., Dias R., Dourado M.C.N., Conde-Sala J.L. (2016). Factors associated with caregiver burden: Comparative study between Brazilian and Spanish caregivers of patients with Alzheimer’s disease (AD). Int. Psychogeriatr..

[25] Cooper C., Katona C., Orrell M., Livingston G. (2008). Coping strategies, anxiety and depression in caregivers of people with Alzheimer’s disease. Int. J. Geriatr. Psychiatry.

[26] Garre-Olmo J., Vilalta-Franch J., Calvo-Perxas L., Turro-Garriga O., Conde-Sala L., Lopez-Pousa S. (2016). A path analysis of patient dependence and caregiver burden in Alzheimer’s disease. Int. Psychogeriatr..

[27] Morgan R.O., Sail K.R., Snow A.L., Davila J.A., Fouladi N.N., Kunik M.E. (2013). Modeling causes of aggressive behavior in patients with dementia. Gerontologist.

[28] Zahir A., Staffaroni A.M., Wickham R.E., Quinn C.M., Sapozhnikova A., Seidman J., Chiong W. (2020). Caregiver “objective attitude” toward patients with neurodegenerative disease: Consequences for caregiver strain and relationship closeness. Aging Ment. Health.

[29] Brodaty H., Woodward M., Boundy K., Ames D., Balshaw R., PRIME Study Group (2014). Prevalence and predictors of burden in caregivers of people with dementia. Am. J. Geriatr. Psychiatry Off. J. Am. Assoc. Geriatr. Psychiatry.

[30] Germain S., Adam S., Olivier C., Cash H., Ousset P.J., Andrieu S., Vellas B., Meulemans T., Reynish E., Salmon E. (2009). Does cognitive impairment influence burden in caregivers of patients with Alzheimer’s disease?. J. Alzheimers Dis. JAD.

[31] Schulz R., McGinnis K.A., Zhang S., Martire L.M., Hebert R.S., Beach S.R., Zdaniuk B., Czaja S.J., Belle S.H. (2008). Dementia patient suffering and caregiver depression. Alzheimer Dis. Assoc. Disord..

[32] Coe N.B., Houtven C.H.V. (2009). Caring for mom and neglecting yourself? The health effects of caring for an elderly parent. Health Econ..

