# Supplementary material for: Making the Invisible Companion of People with Dementia Visible in Economic Studies: What Can We Learn from Social Science?
Source: Healthcare (Basel). 2021 Jan 5;9(1):44. doi: 10.3390/healthcare9010044 (PMC7824847; doi:10.3390/healthcare9010044)
Supplement: Supplementary file 1 [file healthcare-09-00044-s001.pdf]

Web Appendix

Search terms and queries

Database	Search queries
PubMed	((((("Dementia"[Mesh]) AND ("Cross sectional" OR "Cross section" OR longitudinal OR panel)) AND (association OR regression OR correlation)) AND (burden OR stress OR well-being OR "quality of life" OR health)) AND (memory OR cognition OR "Cognitive Impairment" OR function OR behavior OR behaviour OR psychological OR disturbance OR "quality of life" OR health)) AND (caregiver OR career OR caretaker)
EMBASE	('caregiver':ti,ab,kw OR 'carer':ti,ab,kw OR 'caretaker':ti,ab,kw) AND ('memory':ti,ab,kw OR 'cognition':ti,ab,kw OR 'cognitive impairment':ti,ab,kw OR 'function':ti,ab,kw OR 'behavior':ti,ab,kw OR 'behaviour':ti,ab,kw OR 'psychological':ti,ab,kw OR 'quality of life':ti,ab,kw OR 'health':ti,ab,kw) AND ('burden':ti,ab,kw OR 'stress':ti,ab,kw OR 'quality of life':ti,ab,kw OR 'health':ti,ab,kw) AND ('association':ti,ab,kw OR 'regression':ti,ab,kw OR 'correlation':ti,ab,kw OR 'prediction':ti,ab,kw) AND ('cross sectional':ti,ab,kw OR 'cross section':ti,ab,kw OR 'longitudinal':ti,ab,kw) AND ('dementia':ti,ab,kw OR 'alzheimer disease':ti,ab,kw)
All other databases and sources	Caregiver OR Carer OR Caretaker<br>Memory OR Cognition OR "Cognitive Impairment" OR Function OR Behavior OR Behaviour OR Psychological OR Disturbance OR "quality of life" OR health<br>Burden OR Stress OR well-being OR "quality of life" OR health<br>Association OR Regression OR Correlation<br>"Cross sectional" OR "Cross section" OR Longitudinal OR Panel<br>"Dementia"[Mesh]

List of studies included in the scoping review

[1] J. M. Argimon, E. Limon, J. Vila, and C. Cabezas, 'Health-related quality-of-life of care-givers as a predictor of nursing-home placement of patients with dementia', *Alzheimer Dis Assoc Disord*, vol. 19, no. 1, pp. 41–44, Jan. 2005.

[2] G. Berger, T. Bernhardt, E. Weimer, J. Peters, T. Kratzsch, and L. Frolich, 'Longitudinal study on the relationship between symptomatology of dementia and levels of subjective burden and depression among family caregivers in memory clinic patients', *J Geriatr Psychiatry Neurol*, vol. 18, no. 3, pp. 119–128, 2005, doi: 10.1177/0891988704273375.

[3] D. W. Gilley, J. J. McCann, J. L. Bienias, and D. A. Evans, 'Caregiver psychological adjustment and institutionalization of persons with Alzheimer's disease.', *J Aging Health*, vol. 17, no. 2, pp. 172–189, Apr. 2005, doi: 10.1177/0898264304274252.

[4] M. Takahashi, K. Tanaka, and H. Miyaoka, 'Depression and associated factors of informal caregivers versus professional caregivers of demented patients', *Psychiatry Clin Neurosci*, vol. 59, no. 4, pp. 473–480, undefined 2005, doi: 10.1111/j.1440-1819.2005.01401.x.

[5] P. G. Serrano-Aguilar, J. Lopez-Bastida, and V. Yanes-Lopez, 'Impact on Health-Related Quality of Life and

Perceived Burden of Informal Caregivers of Individuals with Alzheimer's Disease', *Neuroepidemiology*, vol. 27, no. 3, pp. 136–142, Oct. 2006, doi: 10.1159/000095760.

[6] K. M. Sink, K. E. Covinsky, D. E. Barnes, R. J. Newcomer, and K. Yaffe, 'Caregiver characteristics are associated with neuropsychiatric symptoms of dementia', *J Am Geriatr Soc*, vol. 54, no. 5, pp. 796–803, undefined 2006, doi: 10.1111/j.1532-5415.2006.00697.x.

[7] N. Matsumoto *et al.*, 'Caregiver burden associated with behavioral and psychological symptoms of dementia in elderly people in the local community', *Dement Geriatr Cogn Disord*, vol. 23, no. 4, pp. 219–224, 2007, doi: 10.1159/000099472.

[8] P. Campbell *et al.*, 'Determinants of burden in those who care for someone with dementia', *Int J Geriatr Psychiatry*, vol. 23, no. 10, pp. 1078–1085, undefined 2008, doi: 10.1002/gps.2071.

[9] C. Cooper, C. Katona, M. Orrell, and G. Livingston, 'Coping strategies, anxiety and depression in caregivers of people with Alzheimer's disease', *Int. J. Geriat. Psychiatry*, vol. 23, no. 9, pp. 929–936, Sep. 2008, doi: 10.1002/gps.2007.

[10] Y. Hirakawa, M. Kuzuya, H. Enoki, J. Hasegawa, and A. Iguch, 'Caregiver burden among Japanese informal caregivers of cognitively impaired elderly in community settings', *Arch Gerontol Geriatr*, vol. 46, no. 3, pp. 367–374, May 2008, doi: 10.1016/j.archger.2007.05.011.

[11] R. Schulz *et al.*, 'Dementia patient suffering and caregiver depression', *Alzheimer Dis Assoc Disord*, vol. 22, no. 2, pp. 170–176, Apr. 2008, doi: 10.1097/WAD.0b013e31816653cc.

[12] S. Germain *et al.*, 'Does cognitive impairment influence burden in caregivers of patients with Alzheimer's disease?', *J Alzheimers Dis*, vol. 17, no. 1, pp. 105–114, 2009, doi: 10.3233/JAD-2009-1016.

[13] M. C. Norton *et al.*, 'Caregiver–Recipient Closeness and Symptom Progression in Alzheimer Disease. The Cache County Dementia Progression Study', *J Gerontol B Psychol Sci Soc Sci*, vol. 64B, no. 5, pp. 560–568, 2009, doi: 10.1093/geronb/gbp052.

[14] C. J. M. Scholzel-Dorenbos, I. Draskovic, M. J. Vernooij-Dassen, and M. G. M. Olde Rikkert, 'Quality of life and burden of spouses of Alzheimer disease patients', *Alzheimer Dis Assoc Disord*, vol. 23, no. 2, pp. 171–177, Apr. 2009.

[15] J. L. Conde-Sala, J. Garre-Olmo, O. Turró-Garriga, J. Vilalta-Franch, and S. López-Pousa, 'Quality of Life of Patients with Alzheimer's Disease: Differential Perceptions between Spouse and Adult Child Caregivers', *Dementia and Geriatric Cognitive Disorders*, vol. 29, no. 2, pp. 97–108, 2010, doi: 10.1159/000272423.

[16] J. L. Conde-Sala, J. Garre-Olmo, O. Turro-Garriga, J. Vilalta-Franch, and S. Lopez-Pousa, 'Differential features of burden between spouse and adult-child caregivers of patients with Alzheimer's disease: an exploratory comparative design.', *Int J Nurs Stud*, vol. 47, no. 10, pp. 1262–1273, Oct. 2010, doi: 10.1016/j.ijnurstu.2010.03.001.

[17] S. Mohamed, R. Rosenheck, C. G. Lyketsos, and L. S. Schneider, 'Caregiver burden in alzheimer disease: Cross-sectional and longitudinal patient correlates', *Am. J. Geriatr. Psychiatry*, vol. 18, no. 10, pp. 917–927, 2010, doi: 10.1097/JGP.0b013e3181d5745d.

[18] C. A. Yeager, L. A. Hyer, B. Hobbs, and A. C. Coyne, 'Alzheimer's Disease and Vascular Dementia: The Complex Relationship between Diagnosis and Caregiver Burden', *Issues in Mental Health Nursing*, vol. 31, no. 6, pp. 376–384, 2010, doi: 10.3109/01612840903434589.

[19] J. M. Garcia-Alberca, J. P. Lara, and M. L. Berthier, 'Anxiety and depression in caregivers are associated with patient and caregiver characteristics in Alzheimer's disease', *Int J Psychiatry Med*, vol. 41, no. 1, pp. 57–69, 2011, doi: 10.2190/PM.41.1.f.

[20] A. A. Mougias, A. Politis, C. G. Lyketsos, and V. G. Mavreas, 'Quality of life in dementia patients in Athens, Greece: predictive factors and the role of caregiver-related factors', *Int Psychogeriatr*, vol. 23, no. 3, pp. 395–403, undefined 2011, doi: 10.1017/S1041610210001262.

[21] I. Contador, B. Fernández-Calvo, D. L. Palenzuela, S. Miguéis, and F. Ramos, 'Prediction of burden in family caregivers of patients with dementia: a perspective of optimism based on generalized expectancies of control', *Aging Ment Health*, vol. 16, no. 6, pp. 675–682, 2012, doi: 10.1080/13607863.2012.684666.

[22] M. Gomez-Gallego, J. Gomez-Amor, and J. Gomez-Garcia, 'Determinants of quality of life in Alzheimer's disease: perspective of patients, informal caregivers, and professional caregivers.', *Int Psychogeriatr*, vol. 24, no. 11, pp. 1805–1815, Nov. 2012, doi: 10.1017/S1041610212001081.

[23] S.-S. Huang, M.-C. Lee, Y.-C. Liao, W.-F. Wang, and T.-J. Lai, 'Caregiver burden associated with behavioral and psychological symptoms of dementia (BPSD) in Taiwanese elderly', *Arch Gerontol Geriatr*, vol. 55, no. 1, pp. 55–59, Jul. 2012, doi: 10.1016/j.archger.2011.04.009.

[24] K. Eska, E. Graessel, C. Donath, L. Schwarzkopf, J. Lauterberg, and R. Holle, 'Predictors of Institutionalization

of Dementia Patients in Mild and Moderate Stages: A 4-Year Prospective Analysis', *Dement Geriatr Cogn Dis Extra*, vol. 3, no. 1, pp. 426–445, Nov. 2013, doi: 10.1159/000355079.

[25] R. O. Morgan, K. R. Sail, A. L. Snow, J. A. Davila, N. N. Fouladi, and M. E. Kunik, 'Modeling causes of aggressive behavior in patients with dementia', *Gerontologist*, vol. 53, no. 5, pp. 738–747, undefined 2013, doi: 10.1093/geront/gns129.

[26] K. Ornstein, J. E. Gaugler, D. P. Devanand, N. Scarmeas, C. Zhu, and Y. Stern, 'The differential impact of unique behavioral and psychological symptoms for the dementia caregiver: How and why do patients' individual symptom clusters impact caregiver depressive symptoms?', *Am. J. Geriatr. Psychiatry*, vol. 21, no. 12, pp. 1277–1286, 2013, doi: 10.1016/j.jagp.2013.01.062.

[27] R. Rosdinom, M. Z. N. Zarina, M. S. Zanariah, M. Marhani, and W. Suzaily, 'Behavioural and psychological symptoms of dementia, cognitive impairment and caregiver burden in patients with dementia', *Prev Med*, vol. 57 Suppl, pp. S67-69, 2013, doi: 10.1016/j.ypmed.2012.12.025.

[28] I. Abdollahpour, S. Nedjat, M. Noroozian, Y. Salimi, and R. Majdzadeh, 'Caregiver burden: the strongest predictor of self-rated health in caregivers of patients with dementia.', *J Geriatr Psychiatry Neurol*, vol. 27, no. 3, pp. 172–180, Sep. 2014, doi: 10.1177/0891988714524627.

[29] H. Brodaty, M. Woodward, K. Boundy, D. Ames, R. Balshaw, and Prime Study Group, 'Prevalence and predictors of burden in caregivers of people with dementia', *Am J Geriatr Psychiatry*, vol. 22, no. 8, pp. 756–765, 2014, doi: 10.1016/j.jagp.2013.05.004.

[30] J. L. Conde-Sala, O. Turro-Garriga, L. Calvo-Perxas, J. Vilalta-Franch, S. Lopez-Pousa, and J. Garre-Olmo, 'Three-year trajectories of caregiver burden in Alzheimer's disease', *J Alzheimers Dis*, vol. 42, no. 2, pp. 623–633, 2014, doi: 10.3233/JAD-140360.

[31] L. N. Gitlin, N. Hodgson, C. V. Piersol, E. Hess, and W. W. Hauck, 'Correlates of quality of life for individuals with dementia living at home: The role of home environment, caregiver, and patient-related characteristics', *Am. J. Geriatr. Psychiatry*, vol. 22, no. 6, pp. 587–597, 2014, doi: 10.1016/j.jagp.2012.11.005.

[32] J. M. Haro *et al.*, 'Analysis of burden in caregivers of people with Alzheimer's disease using self-report and supervision hours.', *J Nutr Health Aging*, vol. 18, no. 7, pp. 677–684, Jul. 2014, doi: 10.1007/s12603-014-0036-0.

[33] N. Hasegawa *et al.*, 'Patient-related factors associated with depressive state in caregivers of patients with dementia at home', *J Am Med Dir Assoc*, vol. 15, no. 5, p. 371.e15–18, 2014, doi: 10.1016/j.jamda.2014.02.007.

[34] M. Kamiya, T. Sakurai, N. Ogama, Y. Maki, and K. Toba, 'Factors associated with increased caregivers' burden in several cognitive stages of Alzheimer's disease', *Geriatr Gerontol Int*, vol. 14 Suppl 2, pp. 45–55, 2014, doi: 10.1111/ggi.12260.

[35] H. S. Kang *et al.*, 'Factors associated with caregiver burden in patients with Alzheimer's disease', *Psychiatr. Invest.*, vol. 11, no. 2, pp. 152–159, 2014, doi: 10.4306/pi.2014.11.2.152.

[36] C. Reed *et al.*, 'Caregiver Burden in Alzheimer's Disease: Differential Associations in Adult-Child and Spousal Caregivers in the GERAS Observational Study', *Dementia and Geriatric Cognitive Disorders Extra*, vol. 4, no. 1, pp. 51–64, Feb. 2014, doi: 10.1159/000358234.

[37] I. Abdollahpour, S. Nedjat, Y. Salimi, M. Noroozian, and R. Majdzadeh, 'Which variable is the strongest adjusted predictor of quality of life in caregivers of patients with dementia?', *Psychogeriatrics*, vol. 15, no. 1, pp. 51–57, Mar. 2015, doi: 10.1111/psyg.12094.

[38] M. C. Alvira *et al.*, 'The association between positive-negative reactions of informal caregivers of people with dementia and health outcomes in eight European countries: a cross-sectional study.', *J Adv Nurs*, vol. 71, no. 6, pp. 1417–1434, Jun. 2015, doi: 10.1111/jan.12528.

[39] P. Bremer *et al.*, 'Informal dementia care: Consequences for caregivers' health and health care use in 8 European countries', *Health Policy*, vol. 119, no. 11, pp. 1459–1471, 2015, doi: 10.1016/j.healthpol.2015.09.014.

[40] G. D'Onofrio *et al.*, 'Caregiver burden characterization in patients with Alzheimer's disease or vascular dementia', *Int J Geriatr Psychiatry*, vol. 30, no. 9, pp. 891–899, 2015, doi: 10.1002/gps.4232.

[41] S.-S. Huang, Y.-C. Liao, and W.-F. Wang, 'Association between caregiver depression and individual behavioral and psychological symptoms of dementia in Taiwanese patients.', *Asia Pac Psychiatry*, vol. 7, no. 3, pp. 251–259, Sep. 2015, doi: 10.1111/appy.12175.

[42] Q. Lou, S. Liu, Y. R. Huo, M. Liu, S. Liu, and Y. Ji, 'Comprehensive analysis of patient and caregiver predictors for caregiver burden, anxiety and depression in Alzheimer's disease', *J Clin Nurs*, vol. 24, no. 17–18, pp. 2668–2678, 2015, doi: 10.1111/jocn.12870.

[43] M. M. L. Nogueira *et al.*, 'Spouse-caregivers' quality of life in Alzheimer's disease', *Int Psychogeriatr*, vol. 27, no. 5, pp. 837–845, undefined 2015, doi: 10.1017/S1041610214002646.

[44] H. Yu, X. Wang, R. He, R. Liang, and L. Zhou, 'Measuring the Caregiver Burden of Caring for Community-

Residing People with Alzheimer's Disease', *PLoS One*, vol. 10, no. 7, p. e0132168, 2015, doi: 10.1371/journal.pone.0132168.
[45] R. Dias *et al.*, 'Caregivers' resilience is independent from the clinical symptoms of dementia', *Arq Neuropsiquiatr*, vol. 74, no. 12, pp. 967–973, undefined 2016, doi: 10.1590/0004-282X20160162.
[46] J. Garre-Olmo, J. Vilalta-Franch, L. Calvo-Perxas, O. Turro-Garriga, L. Conde-Sala, and S. Lopez-Pousa, 'A path analysis of patient dependence and caregiver burden in Alzheimer's disease', *Int Psychogeriatr*, vol. 28, no. 7, pp. 1133–1141, undefined 2016, doi: 10.1017/S1041610216000223.
[47] A. Hausler, A. Sanchez, P. Gellert, F. Deeken, M. A. Rapp, and J. Nordheim, 'Perceived stress and quality of life in dementia patients and their caregiving spouses: does dyadic coping matter?', *Int Psychogeriatr*, vol. 28, no. 11, pp. 1857–1866, Nov. 2016, doi: 10.1017/S1041610216001046.
[48] M. F. B. Sousa, R. L. Santos, O. Turró-Garriga, R. Dias, M. C. N. Dourado, and J. L. Conde-Sala, 'Factors associated with caregiver burden: comparative study between Brazilian and Spanish caregivers of patients with Alzheimer's disease (AD)', *Int Psychogeriatr*, vol. 28, no. 8, pp. 1363–1374, 2016, doi: 10.1017/S1041610216000508.
[49] L. B. Storti, D. T. Quintino, N. M. Silva, L. Kusumota, and S. Marques, 'Neuropsychiatric symptoms of the elderly with Alzheimer's disease and the family caregivers' distress', *Rev Lat Am Enfermagem*, vol. 24, p. e2751, Aug. 2016, doi: 10.1590/1518-8345.0580.2751.
[50] T. H. Valimaki, J. A. Martikainen, K. Hongisto, S. Vaatainen, H. Sintonen, and A. M. Koivisto, 'Impact of Alzheimer's disease on the family caregiver's long-term quality of life: results from an ALSOVA follow-up study', *Qual Life Res*, vol. 25, no. 3, pp. 687–697, undefined 2016, doi: 10.1007/s11136-015-1100-x.
[51] H. Bjorge, K. Kvaal, M. C. Smastuen, and I. Ulstein, 'Relationship Quality and Distress in Caregivers of Persons With Dementia: A Cross-Sectional Study', *Am J Alzheimers Dis Other Demen*, vol. 32, no. 3, pp. 157–165, undefined 2017, doi: 10.1177/1533317517691121.
[52] S. S. W. Choi, C. Budhathoki, and L. N. Gitlin, 'Co-Occurrence and Predictors of Three Commonly Occurring Behavioral Symptoms in Dementia: Agitation, Aggression, and Rejection of Care', *Am J Geriatr Psychiatry*, vol. 25, no. 5, pp. 459–468, undefined 2017, doi: 10.1016/j.jagp.2016.10.013.
[53] S. Liu *et al.*, 'Caregiver burden and prevalence of depression, anxiety and sleep disturbances in Alzheimer's disease caregivers in China.', *J Clin Nurs*, vol. 26, no. 9–10, pp. 1291–1300, May 2017, doi: 10.1111/jocn.13601.
[54] Y. Liu, D. M. Almeida, M. J. Rovine, and S. H. Zarit, 'Care Transitions and Adult Day Services Moderate the Longitudinal Links between Stress Biomarkers and Family Caregivers' Functional Health', *Gerontology*, vol. 63, no. 6, pp. 538–549, 2017, doi: 10.1159/000475557.
[55] M. Torrisi, M. C. De Cola, A. Marra, R. De Luca, P. Bramanti, and R. S. Calabrò, 'Neuropsychiatric symptoms in dementia may predict caregiver burden: a Sicilian exploratory study', *Psychogeriatrics*, vol. 17, no. 2, pp. 103–107, Mar. 2017, doi: 10.1111/psyg.12197.
[56] C.-L. Wang, Y.-I. L. Shyu, J.-Y. Wang, and C.-H. Lu, 'Progressive compensatory symbiosis: spouse caregiver experiences of caring for persons with dementia in Taiwan', *Aging & Mental Health*, vol. 21, no. 3, pp. 241–252, Mar. 2017, doi: 10.1080/13607863.2015.1081148.
[57] D. K. W. Young, P. Y. N. Ng, and T. Kwok, 'Predictors of the health-related quality of life of Chinese people with major neurocognitive disorders and their caregivers: The roles of self-esteem and caregiver's burden', *Geriatrics and Gerontology International*, vol. 17, no. 12, pp. 2319–2328, 2017, doi: 10.1111/ggi.13065.
[58] J. M. Aravena, C. Albala, and L. N. Gitlin, 'Measuring change in perceived well-being of family caregivers: validation of the Spanish version of the Perceived Change Index (PCI-S) in Chilean dementia caregivers', *International Journal of Geriatric Psychiatry*, vol. 33, no. 1, pp. e120–e130, 2018, doi: 10.1002/gps.4734.
[59] L. L. Delfino, R. S. Komatsu, C. Komatsu, A. L. Neri, and M. Cachioni, 'Path analysis of caregiver characteristics and neuropsychiatric symptoms in Alzheimer's disease patients', *Geriatr Gerontol Int*, vol. 18, no. 8, pp. 1177–1182, Aug. 2018, doi: 10.1111/ggi.13437.
[60] I. Hallikainen, A. M. Koivisto, and T. Välimäki, 'The influence of the individual neuropsychiatric symptoms of people with Alzheimer disease on family caregiver distress—A longitudinal ALSOVA study', *International Journal of Geriatric Psychiatry*, vol. 33, no. 9, pp. 1207–1212, 2018, doi: 10.1002/gps.4911.
[61] H. Y. Liu and L. H. Huang, 'The relationship between family functioning and caregiving appraisal of dementia family caregivers: caregiving self-efficacy as a mediator', *Aging Ment Health*, vol. 22, no. 4, pp. 558–567, Apr. 2018, doi: 10.1080/13607863.2016.1269148.
[62] W. Montgomery, A. Goren, K. Kahle-Wrobleski, T. Nakamura, and K. Ueda, 'Alzheimer's disease severity and its association with patient and caregiver quality of life in Japan: results of a community-based survey', *BMC Geriatr*, vol. 18, no. 1, p. 141, Jun. 2018, doi: 10.1186/s12877-018-0831-2.

[63] S. Vatter, K. R. McDonald, E. Stanmore, L. Clare, and I. Leroi, 'Multidimensional Care Burden in Parkinson-Related Dementia', *J Geriatr Psychiatry Neurol*, vol. 31, no. 6, pp. 319–328, Nov. 2018, doi: 10.1177/0891988718802104.

[64] H. Xue, J. Zhai, R. He, L. Zhou, R. Liang, and H. Yu, 'Moderating role of positive aspects of caregiving in the relationship between depression in persons with Alzheimer's disease and caregiver burden', *Psychiatry Res*, vol. 261, pp. 400–405, Mar. 2018, doi: 10.1016/j.psychres.2017.12.088.

[65] A. D. Baharudin, N. C. Din, P. Subramaniam, and R. Razali, 'The associations between behavioral-psychological symptoms of dementia (BPSD) and coping strategy, burden of care and personality style among low-income caregivers of patients with dementia', *BMC Public Health*, vol. 19, no. Suppl 4, p. 447, Jun. 2019, doi: 10.1186/s12889-019-6868-0.

[66] S. S. W. Choi, C. Budhathoki, and L. N. Gitlin, 'Impact of three dementia-related behaviors on caregiver depression: The role of rejection of care, aggression, and agitation', *Int J Geriatr Psychiatry*, vol. 34, no. 7, pp. 966–973, Jul. 2019, doi: 10.1002/gps.5097.

[67] E. L. Guterman *et al.*, 'Association between Caregiver Depression and Emergency Department Use among Patients with Dementia', *JAMA Neurology*, vol. 76, no. 10, pp. 1166–1173, 2019, doi: 10.1001/jamaneurol.2019.1820.

[68] R. Kawaharada, T. Sugimoto, N. Matsuda, Y. Tsuboi, T. Sakurai, and R. Ono, 'Impact of loss of independence in basic activities of daily living on caregiver burden in patients with Alzheimer's disease: A retrospective cohort study', *Geriatr Gerontol Int*, vol. 19, no. 12, pp. 1243–1247, Dec. 2019, doi: 10.1111/ggi.13803.

[69] C. Y. Lin, P. Y. Shih, and L. E. Ku, 'Activities of daily living function and neuropsychiatric symptoms of people with dementia and caregiver burden: The mediating role of caregiving hours', *Arch Gerontol Geriatr*, vol. 81, pp. 25–30, Apr. 2019, doi: 10.1016/j.archger.2018.11.009.

[70] H. Liu, B. Fang, J. Chan, and G. Chen, 'The relationship between comorbidities in dementia patients and burden on adult-child primary caregivers: Does having a secondary caregiver matter?', *Int J Ment Health Nurs*, vol. 28, no. 6, pp. 1306–1317, Dec. 2019, doi: 10.1111/inm.12640.

[71] M. J. Marques *et al.*, 'Relationship quality and sense of coherence in dementia: Results of a European cohort study', *Int J Geriatr Psychiatry*, vol. 34, no. 5, pp. 745–755, May 2019, doi: 10.1002/gps.5082.

[72] L. M. Miller, J. A. Kaye, K. S. Lyons, C. S. Lee, C. J. Whitlatch, and M. S. Caserta, 'Well-being in dementia: a cross-sectional dyadic study of the impact of multiple dimensions of strain on persons living with dementia and their family care partners', *Int Psychogeriatr*, vol. 31, no. 5, pp. 617–626, May 2019, doi: 10.1017/s104161021800203x.

[73] K. Nikzad-Terhune, J. E. Gaugler, and J. Jacobs-Lawson, 'Dementia Caregiving Outcomes: The Impact of Caregiving Onset, Cognitive Impairment and Behavioral Problems', *J Gerontol Soc Work*, vol. 62, no. 5, pp. 543–563, Jul. 2019, doi: 10.1080/01634372.2019.1625993.

[74] S. Okuda, J. Tetsuka, K. Takahashi, Y. Toda, T. Kubo, and S. Tokita, 'Association between sleep disturbance in Alzheimer's disease patients and burden on and health status of their caregivers', *J Neurol*, vol. 266, no. 6, pp. 1490–1500, Jun. 2019, doi: 10.1007/s00415-019-09286-0.

[75] C. A. Polenick, C. W. Sherman, K. S. Birditt, S. H. Zarit, and H. C. Kales, 'Purpose in Life Among Family Care Partners Managing Dementia: Links to Caregiving Gains', *Gerontologist*, vol. 59, no. 5, pp. e424–e432, Sep. 2019, doi: 10.1093/geront/gny063.

[76] R. Rodríguez-González, A. E. Martínez-Santos, D. Facal, J. Hermida-Porto, C. Rodríguez-Abad, and M. Gandoy-Crego, 'Pattern of care and impact on family caregivers of people with dementia from a nursing care model perspective', *International Psychogeriatrics*, vol. 31, pp. 139–140, 2019, doi: 10.1017/S1041610219001339.

[77] P. Ruisoto, I. Contador, B. Fernández-Calvo, D. Palenzuela, and F. Ramos, 'Exploring the association between optimism and quality of life among informal caregivers of persons with dementia', *Int Psychogeriatr*, vol. 31, no. 3, pp. 309–315, Mar. 2019, doi: 10.1017/s104161021800090x.

[78] M. Sakka, J. Goto, S. Kita, I. Sato, T. Soejima, and K. Kamibeppu, 'Associations among behavioral and psychological symptoms of dementia, care burden, and family-to-work conflict of employed family caregivers', *Geriatr Gerontol Int*, vol. 19, no. 1, pp. 51–55, Jan. 2019, doi: 10.1111/ggi.13556.

[79] E. K. Vernon *et al.*, 'Caregiver-Care Recipient Relationship Closeness is Associated With Neuropsychiatric Symptoms in Dementia', *Am J Geriatr Psychiatry*, vol. 27, no. 4, pp. 349–359, Apr. 2019, doi: 10.1016/j.jagp.2018.11.010.

[80] F. Yang, M. Ran, and W. Luo, 'Depression of persons with dementia and family caregiver burden: Finding positives in caregiving as a moderator', *Geriatr Gerontol Int*, vol. 19, no. 5, pp. 414–418, May 2019, doi: 10.1111/ggi.13632.

[81] B. Akplnar Söylemez, Ö. Küçükgüçlü, M. A. Akyol, and A. T. Işlk, 'Quality of life and factors affecting it in patients with Alzheimer's disease: A cross-sectional study', *Health and Quality of Life Outcomes*, vol. 18, no. 1, 2020, doi: 10.1186/s12955-020-01554-2.

[82] M. H. Connors, K. Seeher, A. Teixeira-Pinto, M. Woodward, D. Ames, and H. Brodaty, 'Dementia and caregiver burden: A three-year longitudinal study', *Int J Geriatr Psychiatry*, vol. 35, no. 2, pp. 250–258, Feb. 2020, doi: 10.1002/gps.5244.

[83] L. Hvidsten *et al.*, 'Quality of life of family carers of persons with young-onset compared to late-onset dementia', *Aging & mental health*, vol. 24, no. 9, pp. 1394–1401, 2020, doi: 10.1080/13607863.2019.1617245.

[84] L. H. Jütten, R. E. Mark, and M. M. Sitskoorn, 'Predicting self-esteem in informal caregivers of people with dementia: Modifiable and non-modifiable factors', *Aging & mental health*, vol. 24, no. 2, pp. 221–226, 2020, doi: 10.1080/13607863.2018.1531374.

[85] L. Kamalzadeh *et al.*, 'Perceived burden of dementia care, clinical, psychological and demographic characteristics of patients and primary caregivers in Iran', *Applied neuropsychology. Adult*, pp. 1–12, 2020, doi: 10.1080/23279095.2020.1798960.

[86] N. R. S. Kimura *et al.*, 'Young- and Late-Onset Dementia: A Comparative Study of Quality of Life, Burden, and Depressive Symptoms in Caregivers', *Journal of Geriatric Psychiatry and Neurology*, 2020, doi: 10.1177/0891988720933355.

[87] I. Parrotta *et al.*, 'Depression in People With Dementia and Caregiver Outcomes: Results From the European Right Time Place Care Study', *Journal of the American Medical Directors Association*, vol. 21, no. 6, pp. 872-878.e1, 2020, doi: 10.1016/j.jamda.2020.02.023.

[88] J. A. Su and C. C. Chang, 'Association Between Family Caregiver Burden and Affiliate Stigma in the Families of People with Dementia', *Int J Environ Res Public Health*, vol. 17, no. 8, Apr. 2020, doi: 10.3390/ijerph17082772.

[89] L. B. Taranrød, S. Eriksen, I. Pedersen, and Ø. Kirkevold, 'Characteristics, burden of care and quality of life of next of kin of people with dementia attending farm-based day care in Norway: A descriptive cross-sectional study', *Journal of Multidisciplinary Healthcare*, vol. 13, pp. 1363–1373, 2020, doi: 10.2147/JMDH.S268818.

[90] A. Teahan, A. Lafferty, J. Cullinan, G. Fealy, E. O'Shea, and A. Teahan, 'An analysis of carer burden among family carers of people with and without dementia in Ireland', *International Psychogeriatrics*, 2020, doi: 10.1017/S1041610220000769.

[91] V. W. Wang, N. Kandiah, X. Lin, and H. L. Wee, 'Does health-related quality of life in Asian informal caregivers differ between early-onset dementia and late-onset dementia?', *Psychogeriatrics*, vol. 20, no. 5, pp. 608–619, 2020, doi: 10.1111/psyg.12556.

[92] A. Zahir *et al.*, 'Caregiver "objective attitude" toward patients with neurodegenerative disease: Consequences for caregiver strain and relationship closeness', *Aging & mental health*, pp. 1–7, 2020, doi: 10.1080/13607863.2020.1771541.
